# Supplementary material for: Tagitinin A regulates an F-box gene, CPR30, to resist tomato spotted wilt orthotospovirus (TSWV) infection in Nicotiana benthamiana
Source: PLoS One. 2024 Dec 10;19(12):e0315294. doi: 10.1371/journal.pone.0315294 (PMC11630581; doi:10.1371/journal.pone.0315294)

VIGS-NSm

M P1-I-m V-I-m V-S-m P1-S-m

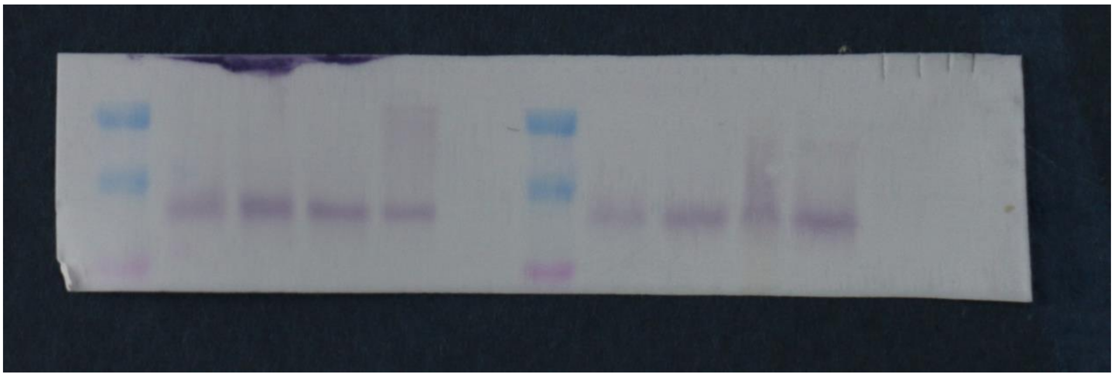

Actin

M P1-I-m V-I-m V-S-m P1-S-m

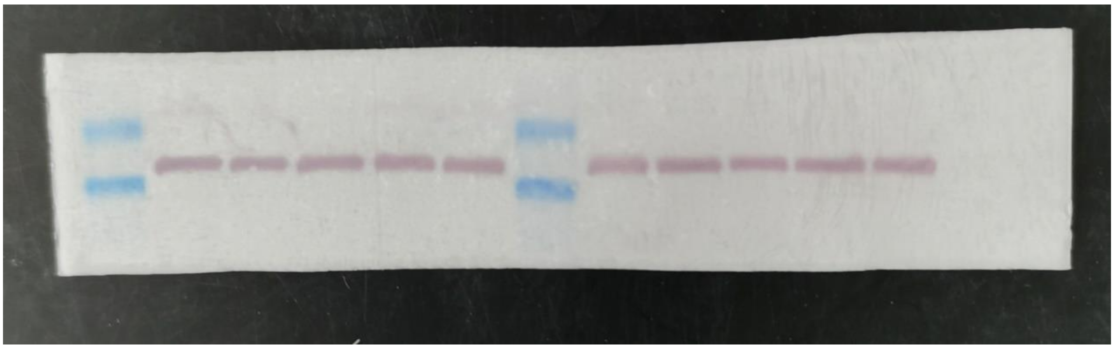

VIGS-NSs

M P1-I-s V-Is V-S-s P1-S-s

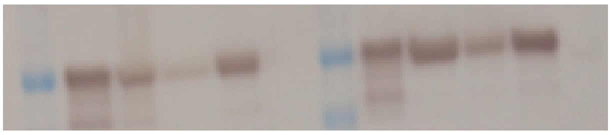

Actin

M P1-I-s V-Is V-S-s P1-S-s

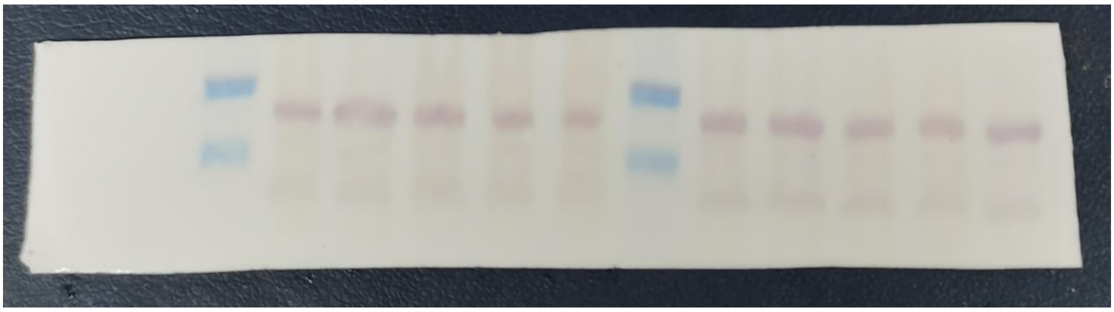

Overexpression-NSm

M O-I-m P2-I-m P2-S-m O-S-m

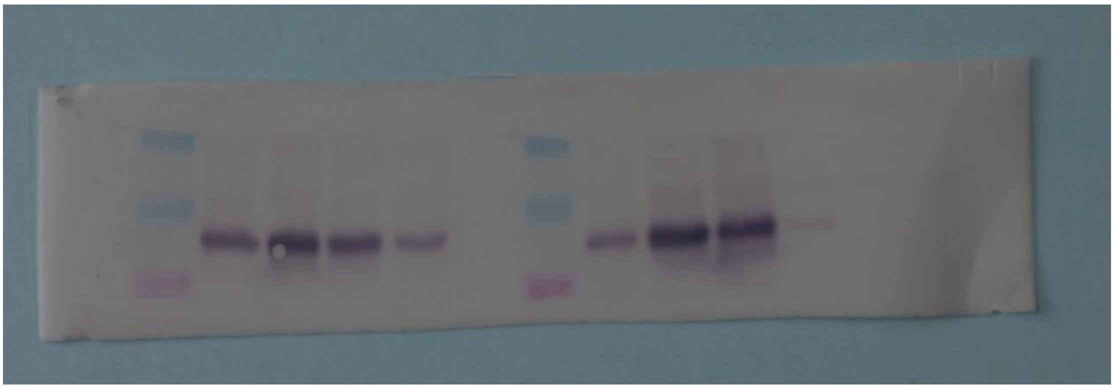

Actin

M O-I-m P2-I-m P2-S-m O-S-m

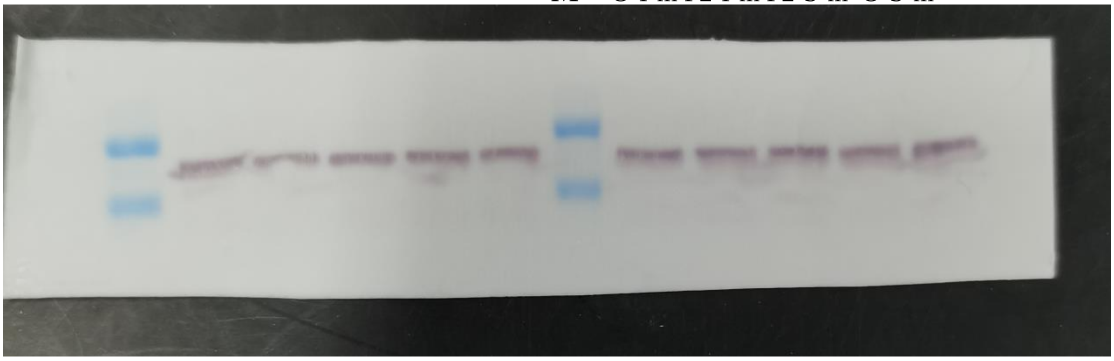

Overexpression-NSs

M P2-I-s O-I-s P2-S-s O-S-s

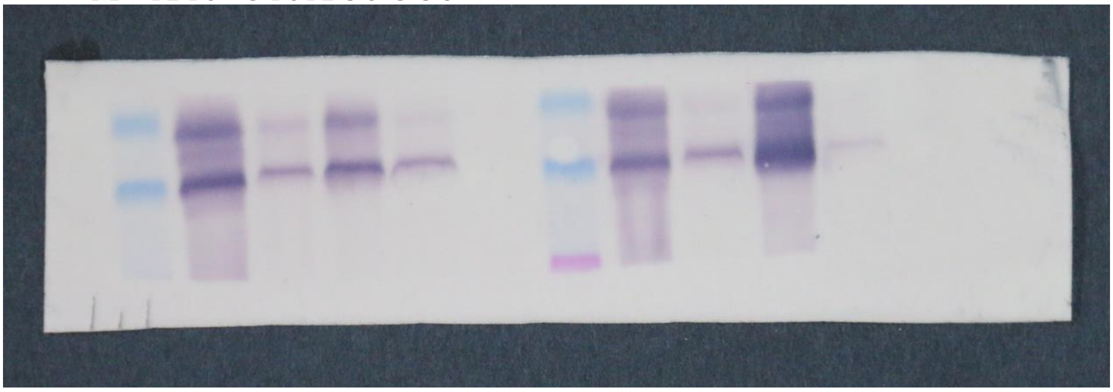

Actin

M P2-I-s O-I-s P2-S-s O-S-s

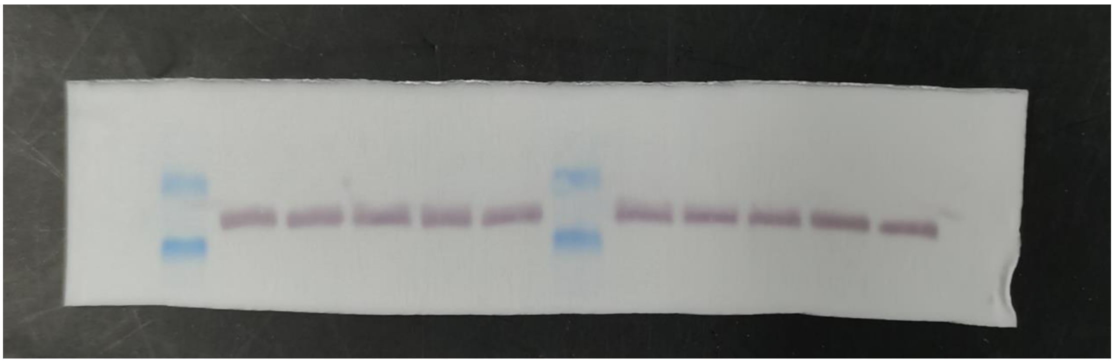

Supplement: S1 Raw images — (PDF) [file pone.0315294.s001.pdf]
