# Supplementary material for: Tagitinin A regulates an F-box gene, CPR30, to resist tomato spotted wilt orthotospovirus (TSWV) infection in Nicotiana benthamiana
Source: PLoS One. 2024 Dec 10;19(12):e0315294. doi: 10.1371/journal.pone.0315294 (PMC11630581; doi:10.1371/journal.pone.0315294)
Supplement: S1 Table — (DOCX) [file pone.0315294.s002.docx]

Supplementary Table 1 The primers used in this study

| Primer name | Sequence(5´-3´) | Base (bp) | Primer function | restriction enzyme | Gene name |
| --- | --- | --- | --- | --- | --- |
| QAt4g22390-F | AAACTGCCCAAATCTTCT | 18 | RT-qPCR |  | *CPR30* |
| QAt4g22390-R | ATTCCTTCATCACCCACA | 18 | RT-qPCR |  |  |
| QAt4g37680-F | CAATGAAGGTCCCAGATA | 18 | RT-qPCR |  | *HTP-4* |
| QAt4g37680-R | CGCTCAGGCAGACAGTTA | 18 | RT-qPCR |  |  |
| QLOC_Os08g37250-F | GCTAACAATCCAACCCTT | 18 | RT-qPCR |  | *PLP2* |
| QLOC_Os08g37250-R | TAGCCAACCTAAGATACCC | 18 | RT-qPCR |  |  |
| QAt1g21910-F | AAGTGGCAGCGGCTCAAT | 18 | RT-qPCR |  | *ERF01* |
| QAt1g21910-R | TCCGACGATGACGATGAA | 18 | RT-qPCR |  |  |
| QAt3g53200-F | GGGAAATAGATGGTCAAG | 18 | RT-qPCR |  | *MYB48* |
| QAt3g53200-R | GGTAATGGGCATAAAGTC | 18 | RT-qPCR |  |  |
| QAt1g70830-F | GGGTCTGAAAGGCAAGTT | 18 | RT-qPCR |  | *KIX2* |
| QAt1g70830-R | CTTCCTTAACCGTCGTAT | 17 | RT-qPCR |  |  |
| VIGAt4g22390-F | CCCAAGCTTTCCGTGTTTGTCCAGTTT | 27 | VIGS | *Hind III* | *CPR30* |
| VIGAt4g22390-R | CGGGATCCTCAGGTGCGTCACAGAGC | 26 | VIGS | *BamH I* |  |
| QTSWV-NSs-F | GTCTCCTGCTCAGCTCCATTC | 22 | qRT-PCR |  | TSWV-*NSs* |
| QTSWV-NSs-R | TTTCTTGGAGCTGGAATCGGT | 22 | qRT-PCR |  |  |
| QTSWV-NSm-F | TGGGTCTGCCCCACTATACC | 21 | qRT-PCR |  | TSWV-*NSm* |
| QTSWV-NSm-R | CAGATGGCATGTTGGGATCG | 21 | qRT-PCR |  |  |
| SNAt4g22390-F | CCGGAATTCATGAGTGATCAAACAACAACCA | 31 | Over-expression | *EcoR I* | *CPR30* |
| SNAt4g22390-R | CGCGGATCCGCAAATCCAATTCCATTCTGGT | 31 | Over-expression | *BamH I* |  |
| ADAt4g22390-F | CCGGAATTCATGAGTGATCAAACAACAACCA | 31 | Y2H | *EcoR I* | *CPR30* |
| ADAt4g22390-R | CGCGGATCCGCAAATCCAATTCCATTCTGGT | 31 | Y2H | *BamH I* |  |
| BDTSWV-NSs-F | CCGGAATTC ATGTCTTCAAGTGTTTATGAGTCG | 34 | Y2H | *EcoR I* | TSWV-*NSs* |
| BDTSWV-NSs-R | CGGGATCCTTATTTTGATCCTGAAGCATATGC | 32 | Y2H | *BamH I* |  |
| BDTSWV-NSm-F | AGGACCTGCATATGGCCATGGATGTTGACTTTTTTTGGTAATAAG | 45 | Y2H | *Nco I* | TSWV-*NSm* |
| BDTSWV-NSm-R | ATGCGGCCGCTGCAGGTCGACTCATATCTCATCAAAAGATAACTGA | 46 | Y2H | *Sal I* |  |
| B-T-NSm-F | CGGGATCCATGTTGACTTTTTTTGGTAATAAG | 31 | BiFC | *BamH I* | TSWV-*NSm* |
| B-T-NSm-R | ACGCGTCGACTATCTCATCAAAAGATAACTGAGC | 35 | BiFC | *Sal I* |  |
| B-T-NSs-F | CGGGATCC ATGTCTTCAAGTGTTTATGAGTCG | 33 | BiFC | *BamH I* | TSWV-*NSs* |
| B-T-NSs-R | ACGCGTCGACTTATTTTGATCCTGAAGCATATGC | 35 | BiFC | *Sal I* |  |
| B-At4g22390-F | CGGGATCCCATGAGTGATCAAACAACAACCA | 33 | BiFC | *BamH I* | *CPR30* |
| B-At4g22390-R | ACGCGTCGACGCAAATCCAATTCCATTCTGGT | 32 | BiFC | *Sal I* |  |
| L-T-NSm-F | CGGGATCCATGTTGACTTTTTTTGGTAATAAG | 32 | LUC | *BamH I* | TSWV-*NSm* |
| L -T-NSm-R | ACGCGTCGACTATCTCATCAAAAGATAACTGAGC | 34 | LUC | *Sal I* |  |
| L -T-NSs-F | CGGGATCCATGTCTTCAAGTGTTTATGAGTCG | 32 | LUC | *BamH I* | TSWV-*NSs* |
| L -T-NSs-R | ACGCGTCGACTTATTTTGATCCTGAAGCATATGC | 34 | LUC | *Sal I* |  |
| L -At4g22390-F | CGGGATCCCATGAGTGATCAAACAACAACCA | 32 | LUC | *BamH I* | *CPR30* |
| L-At4g22390-R | ACGCGTCGACGCAAATCCAATTCCAATTCTGGT | 33 | LUC | *Sal I* |  |
