# Supplementary material for: Tagitinin A regulates an F-box gene, CPR30, to resist tomato spotted wilt orthotospovirus (TSWV) infection in Nicotiana benthamiana
Source: PLoS One. 2024 Dec 10;19(12):e0315294. doi: 10.1371/journal.pone.0315294 (PMC11630581; doi:10.1371/journal.pone.0315294)
Supplement: Attachment — Submitted filename: Response to Reviewers.docx [file pone.0315294.s003.docx]

Journal Requirements:

1. When submitting your revision, we need you to address these additional requirements.

Please ensure that your manuscript meets PLOS ONE's style requirements, including those for file naming. The PLOS ONE style templates can be found at

<https://journals.plos.org/plosone/s/file?id=wjVg/PLOSOne_formatting_sample_main_body.pdf> and<https://journals.plos.org/plosone/s/file?id=ba62/PLOSOne_formatting_sample_title_authors_affiliations.pdf>

**Answer：**We have ensured that your manuscript meets PLOS ONE's style requirements, including those for file naming

2. Thank you for stating the following financial disclosure: "This work was carried out with supporting from National Natural Science Foundation of China (No.32360639), Yunnan Young & Elite Talents Project (YNWR-QNRC-2020 to JZ, YNWR-QNRC-2022 to LHZ), Yunnan Provincial Natural Science Foundation (No. 202401AS070031)."

Please state what role the funders took in the study. If the funders had no role, please state: "The funders had no role in study design, data collection and analysis, decision to publish, or preparation of the manuscript."

If this statement is not correct you must amend it as needed.

Please include this amended Role of Funder statement in your cover letter; we will change the online submission form on your behalf.

**Answer：**In cover letter, we added the content “This work was supported by National Natural Science Foundation of China (No.32360639), Yunnan Young & Elite Talents Project (YNWR-QNRC-2020 to JZ, YNWR-QNRC-2022 to LHZ), Yunnan Provincial Natural Science Foundation (No. 202401AS070031). The funder of H.L.Z. conceived, designed the experiments, and wrote the manuscript, J.Z. analyzed the data and revised the manuscript. ”

3. Thank you for stating the following in the Acknowledgments Section of your manuscript: "This work was carried out with supporting from National Natural Science Foundation of China (No.32360639), Yunnan Young & Elite Talents Project (YNWR-QNRC-2020 to JZ, YNWR-QNRC-2022 to LHZ), Yunnan Provincial Natural Science Foundation (No. 202401AS070031)."

We note that you have provided funding information that is not currently declared in your Funding Statement. However, funding information should not appear in the Acknowledgments section or other areas of your manuscript. We will only publish funding information present in the Funding Statement section of the online submission form.

Please remove any funding-related text from the manuscript and let us know how you would like to update your Funding Statement. Currently, your Funding Statement reads as follows: "This work was carried out with supporting from National Natural Science Foundation of China (No.32360639), Yunnan Young & Elite Talents Project (YNWR-QNRC-2020 to JZ, YNWR-QNRC-2022 to LHZ), Yunnan Provincial Natural Science Foundation (No. 202401AS070031)."

Please include your amended statements within your cover letter; we will change the online submission form on your behalf.

**Answer：**Line 273-276, the content “This work was carried out with supporting from National Natural Science Foundation of China (No.32360639), Yunnan Young & Elite Talents Project (YNWR-QNRC-2020 to JZ, YNWR-QNRC-2022 to LHZ), Yunnan Provincial Natural Science Foundation (No. 202401AS070031).” has been deleted in the manuscript. In cover letter, the content “This work was carried out with supporting from National Natural Science Foundation of China (No.32360639), Yunnan Young & Elite Talents Project (YNWR-QNRC-2020 to JZ, YNWR-QNRC-2022 to LHZ), Yunnan Provincial Natural Science Foundation (No. 202401AS070031).” Has been changed into “This work was supported by National Natural Science Foundation of China (No.32360639), Yunnan Young & Elite Talents Project (YNWR-QNRC-2020 to JZ, YNWR-QNRC-2022 to LHZ), Yunnan Provincial Natural Science Foundation (No. 202401AS070031). The funder of H.L.Z. conceived, designed the experiments, and wrote the manuscript, J.Z. analyzed the data and revised the manuscript. ”

4. Please provide a complete Data Availability Statement in the submission form, ensuring you include all necessary access information or a reason for why you are unable to make your data freely accessible. If your research concerns only data provided within your submission, please write "All data are in the manuscript and/or supporting information files" as your Data Availability Statement.

**Answer：**Line 271, the word “**Declarations**” has been changed into “**DATA AVAILABILITY** All data were available within this article and its supplementary files. All constructs are available upon request.”

5. PLOS ONE now requires that authors provide the original uncropped and unadjusted images underlying all blot or gel results reported in a submission’s figures or Supporting Information files. This policy and the journal’s other requirements for blot/gel reporting and figure preparation are described in detail at <https://journals.plos.org/plosone/s/figures#loc-blot-and-gel-reporting-requirements> and <https://journals.plos.org/plosone/s/figures#loc-preparing-figures-from-image-files.> When you submit your revised manuscript, please ensure that your figures adhere fully to these guidelines and provide the original underlying images for all blot or gel data reported in your submission. See the following link for instructions on providing the original image data: [https://journals.plos.org/plosone/s/figures#loc-original-images-for-blots-and-gels.](https://journals.plos.org/plosone/s/figures#loc-original-images-for-blots-and-gels.)

In your cover letter, please note whether your blot/gel image data are in Supporting Information or posted at a public data repository, provide the repository URL if relevant, and provide specific details as to which raw blot/gel images, if any, are not available. Email us at [plosone@plos.org](mailto:plosone@plos.org) if you have any questions.

**Answer：**In cover letter, we added the sentence "Our blot/gel image data are in Supporting Information and provide specific details as to which raw blot/gel images".

**Comments to the Author**

1. Is the manuscript technically sound, and do the data support the conclusions?
The manuscript must describe a technically sound piece of scientific research with data that supports the conclusions. Experiments must have been conducted rigorously, with appropriate controls, replication, and sample sizes. The conclusions must be drawn appropriately based on the data presented.

Reviewer #1: Yes；Reviewer #2: Partly；Reviewer #3: Partly；Reviewer #4: Yes

**Answer：**Our experiments have been conducted rigorously, and with appropriate controls, replication, and sample sizes, several experimental details have been added in part of “Materials and methods” and illustrated them in “Changes by changes”.

2. Has the statistical analysis been performed appropriately and rigorously?

Reviewer #1: N/A；Reviewer #2: Yes；Reviewer #3: Yes；Reviewer #4: N/A

**Answer：**We have inserted the part of “Statistical analysis” followed Line158-159, and illustrated them in “Changes by changes”.

3. Have the authors made all data underlying the findings in their manuscript fully available?
The [PLOS Data policy](http://c05y1x9s.r.us-east-2.awstrack.me/L0/http:%2F%2Fwww.plosone.org%2Fstatic%2Fpolicies.action%23sharing/1/010f0191e3efa9b3-1b729b5b-4430-4366-a0d2-0e4a09de0c3c-000000/vTDAV8bYHfaNk6ArSKoUlfA_WrU=176) requires authors to make all data underlying the findings described in their manuscript fully available without restriction, with rare exception (please refer to the Data Availability Statement in the manuscript PDF file). The data should be provided as part of the manuscript or its supporting information, or deposited to a public repository. For example, in addition to summary statistics, the data points behind means, medians and variance measures should be available. If there are restrictions on publicly sharing data—e.g. participant privacy or use of data from a third party—those must be specified.

Reviewer #1: No；Reviewer #2: Yes；Reviewer #3: Yes；Reviewer #4: Yes

**Answer：**All data underlying the findings in our manuscript fully available. Page 10, line 271: the content “Declarations” has been changed into “DATA AVAILABILITY

All data were available within this article and its supplementary files. All constructs are available upon request.”

4. Is the manuscript presented in an intelligible fashion and written in standard English?
PLOS ONE does not copyedit accepted manuscripts, so the language in submitted articles must be clear, correct, and unambiguous. Any typographical or grammatical errors should be corrected at revision, so please note any specific errors here.

Reviewer #1: No；Reviewer #2: No；Reviewer #3: Yes；Reviewer #4: Yes

**Answer：**We have modified the specific errors and the LetPub (www.letpub.com.cn) for its linguistic assistance during the revision of this manuscript.

5. Review Comments to the Author

**Reviewer #1:** Dear Authors,

The findings of the manuscript displays a route map to exploit metabolites of plant origin to mitigate viral diseases. This will provide knowledge on tritrophic interactions. But the paper is with lot of grammar mistakes and English is very poor. The paper can be readable only if it is modified with good and correct English.
Few comments as sample:
(1)Title has to be changed as “Tagitinin A regulating an F-box gene, CPR30 to resist Orthotospovirus tomatomaculae (TSWV) infection
**Answer：**Line 2, the title has been changed into “Tagitinin A regulating regulates an F-box gene, CPR30, to resist tomato spotted wilt orthotospovirus (TSWV) infection in *Nicotiana benthamiana*”**.**

(2)The paper describes all studies only in N. *bethamiana* as model, in that case N.*benthamana* must come in title???
**Answer：**Line 3, inserted the words “in *Nicotiana benthamiana*” followed the word “infection”**.**

(3)Line 33: Give the new name of Tomato spotted wilt orthotospovirus as “Orthotospovirus tomatomaculae, which is a new name assigned, please refer ICTV 2023 (TSWV) belongs to the species Tospovirus and add ICTV link as reference. Then give the old name TSWV inside the manuscript.

**Answer：**Line 33, the sentence “Tomato spotted wilt orthotospovirus (TSWV) belongs to the species Tospovirus, genus Orthotospovirus and family Tospovirdae” has been changed into “Tomato spotted wilt orthotospovirus (TSWV, synonym Orthotospovirus tomatomaculae) belongs to the species Tospovirus, genus Orthotospovirus and family Tospovirdae (ICTV, 2022)”

(4)Line 57-58- Modify the sentence “resistance systemic acquired resistance (SAR)” to get correct meaning

**Answer：**Line 57, deleted the word “resistance”.

(5)Line 60: Change as “However, the biotic products regulating
**Answer：**Line 60, the word “regulates” has been changed into “regulating”

(6)68-69: Grammar mistake. Modify the sentence.

**Answer：**Line 68-69, the sentence “In this study, we described Tag A regulate the F-box protein (CPR30) to interacts with the TSWV-NSm protein and inhibits TSWV infection.” has been changed into “In this study, we found that Tag A regulated the F-box protein (CPR30) interacting with the TSWV-NSm protein and inhibited TSWV infection.”

**Reviewer #2:** Previously, the authors found that Tagitinin A (Tag A) can suppress the expression of NSm (the movement protein) and NSs (the silencing suppressor protein) of tomato spotted wilt virus (TSWV). In this study, the authors investigated the molecular mechanism by which Tag A combats the TSWV using the yeast two-hybrid (Y2H) and virus-induced gene silencing (VIGS) assays. The results showed that TSWV-NSm can induce the expression of the F-box protein (CPR30) gene and interact with its translational product. Silencing of the CPR30 could significantly increase the replication and expression of TSWV-NSm/NSs, whereas over-expression of CPR30 displayed contrary results. These findings show a new mechanism in Tag A mediating CPR30, which mainly acts on NSm protein to prevent TSWV systematic infection. However, the present manuscript is deficient in several respects, including deficiencies in English language proficiency and inaccuracies in text formatting. Therefore, I recommend a Major Revision of the manuscript. Some comments are given below.
(1)The first major issue with the manuscript is the quality of the English. On first reviewing the paper, I made numerous edits/changes to the text and found myself unnecessarily distracted from assessing the paper on scientific grounds. I appreciate that English may not be the first language of the authors, but there is no doubt that the quality of the paper will be affected.

**Answer：**We have improved the English of this paper, and this manuscript also has been linguistic revised and edited by LetPub.

(2)It is recommended that the authors rewrite the sections on Materials and methods and Results to improve the logical coherence between the individual methods and results.

**Answer：**We have been rewrote the sections on Materials and methods and Results, and illustrated them in “Changes by changes”.

(3)The visual representation presented in Figure 4 is not sufficiently clear.

**Answer：**We have been tried our best to adjust the picture, but the visual representation presented in Figure 4 before is the best condition, so we didn't change them, and we provided the original image.

(4)The authors should follow the PLOS ONE Guidelines for Authors and ensure that precise use of punctuation, font, size, line spacing, and paragraph formatting is followed.

**Answer：**We have modified the punctuation, font, size, line spacing, and paragraph formatting of the article follow the PLOS ONE Guidelines for Authors and list them in changes by changes.

(5)Please check all the references and make sure that they conform to acceptable formats of PLOS ONE.

**Answer：**We have modified the references formatting of the article follow the PLOS ONE Guidelines for Authors and list them in changes by changes.

**Reviewer #3:** In this manuscript, the authors identified the effects of biogenic pesticide TagA on tomato spotted wilt orthotospovirus （TSWV）by regulating an F-box gene, CPR30. They indicated that the expression of the CPR30 gene in Tag A-treated leaves was significant higher (2 times) than that the negative control. And the replication of TSWV-NSm/NSs genes and the expression of TSWV-NSm/NSs proteins significantly increased after the CPR30 genewas silenced in protective assays, over-expression of CPR30 showed the contrary tendency. the CPR30 protein only localizes and interacts with TSWV-NSm protein. Overall, this study directly demonstrates the regulatory function of CPR30 during tomato spotted wilt orthotospovirus infection. Otherwise, in current manuscript, about the real relationship among Tag A, CPR30 and resisting TSWV infection may less direct or clear. If the authors have already conducted the TagA treatment with CPR30 expression level (up and down-regulated) together at the same time, to compare the effects of TagA treatment, CPR30 expression level and TagA+CPR30 expression level on spotted wilt orthotospovirus infection, it is suggested to supplement the treatments and the results clear to the manuscript to clarify which is the primary activator and how the reaction each other to spotted wilt orthotospovirus infection.

**Answer：**In result part 2 and 3, when we silenced or over-expressed CPR30 gene, the protective experiment (smeared the Tag A and inoculated the TSWV after 6 h) was performed, the control was performed the protective experiment (smeared the Tag A and inoculated the TSWV after 6 h) only, the compared results could sufficiently explain the real relationship among Tag A, CPR30 and resisting TSWV infection.

**Reviewer #4:** The manuscript is well written but followings point will enhance the quality of manuscript.
(1) Abstract lack novel statement that how it is different from previous research.

**Answer：**Line 17-18, the sentence “Tag A could suppress the expression of NSm (the movement protein) and NSs (the silencing suppressor protein) of TSWV.” has been changed into “Tag A can induce jasmonic acid to suppress gene expression in TSWV.”; Line 18-19, the sentence “However, the underlying molecular mechanism of Tag A-mediated antiviral activity was largely unknown.” was deleted; Line 26-29, the content “Thus, this study reveals a new mechanism in Tag A mediating CPR30 mainly acting on NSm protein to prevent TSWV systematic infection. The present biogenic antiviral molecules will be helpful in development of suitable eco-friendly formulations to mitigate TSWV infection disease in plants.” has been changed into “Thus, this study reveals a new mechanism by which Tag A mediates ubiquitin–protein ligase E3 (CPR30) to interact with NSm inhibite NSm replication and expression, and defend against systemic TSWV infection.”.
(2) Materials and methods should be brief.

**Answer：**We have rewrote the sections on Materials and methods, and illustrated them in “Changes by changes”.

(3)There should be need for the project in introduction showing that how this research is needed in the field of plant pathology.

**Answer：**Line 69, inserted the sentence “The present biogenic antiviral molecules will be helpful in the development of suitable eco-friendly formulations to mitigate TSWV infection in plants.”.

(4) Discussion should be expanded.

Answer：We have expanded the sections on Discussion, and illustrated them in “Changes by changes”.

(5) Please conclude your research and elaborate the future prospect.

**Answer：**Line 270, we inserted the content “Conclusions This study showed that *CPR30* gene expression was two times higher in Tag A-treated leaves than in the negative control. Moreover, the transcription levels of the *NSm* and *NSs* genes and the expression of the NSm and NSs proteins were significantly higher in TRV-CPR30 and significantly lower in CPR30-overexpressing tobacco plants than in the inoculated and systemic leaves of the positive control. Further research showed that CPR30 co-localized and directly interacted with TSWV-NSm. Taken together, our results indicated that Tag A activated CPR30 to induce systemic resistance, inhibiting TSWV gene replication and protein expression and defending against TSWV infection. The results indicated the unique mechanism by which Tag A regulated a plant defense response gene to defend against TSWV infection. Future studies should evaluate this as a tool for developing virus-resistant varieties.” followed the line 270.

6. PLOS authors have the option to publish the peer review history of their article ([what does this mean?](https://c05y1x9s.r.us-east-2.awstrack.me/L0/https:%2F%2Fjournals.plos.org%2Fplosone%2Fs%2Feditorial-and-peer-review-process%23loc-peer-review-history/1/010f0191e3efa9b3-1b729b5b-4430-4366-a0d2-0e4a09de0c3c-000000/mRzS6G6X0mYrBJvIlTvtjqYN2tQ=176)). If published, this will include your full peer review and any attached files.
If you choose “no”, your identity will remain anonymous but your review may still be made public.
**Do you want your identity to be public for this peer review?** For information about this choice, including consent withdrawal, please see our [Privacy Policy](https://c05y1x9s.r.us-east-2.awstrack.me/L0/https:%2F%2Fwww.plos.org%2Fprivacy-policy/1/010f0191e3efa9b3-1b729b5b-4430-4366-a0d2-0e4a09de0c3c-000000/JJztJBIQPtS5j38TjQQaRSldgRc=176).

Reviewer #1: No;Reviewer #2: No;Reviewer #3: No;Reviewer #4: **Yes:** Yasir Iftikhar

**Answer：**We choose “No”.
